# Underdiagnosis and undertreatment of asthma in children: a tertiary hospital's experience

**DOI:** 10.1186/2045-7022-5-S2-P19

**Published:** 2015-03-23

**Authors:** Ioanna Vasilopoulou, Irene Papakonstantopoulou, Katerina Salavoura, Nikoletta Laliotou, Athanasios Kaditis, Vasiliki Gemou-Engesaeth

**Affiliations:** 1 University of Athens, Greece, First Department of Pediatrics, Athens, Greece

## Background

The aim of this study was to outline possible causes of under-diagnosis of asthma and of inadequately defined difficult asthma in children referred to a tertiary center.

## Methods

We studied 82 children (age 2-15y) that were referred to our clinic during 2013-2014 and their history and/or physical examination revealed a clinical suspicion of asthma, according to GINA. Children were evaluated by personal/family history, physical examination, skin prick tests to common allergens, total/specific IgE levels. Lung function tests were carried out where possible. Chest X-ray and sweat test were performed if needed. Children were divided into three groups: children with asthma diagnosed for first time, children with asthma whose symptoms were uncontrolled and children with severe/persistent asthma.

## Results

32/82 children were diagnosed with asthma for the first time in our Unit and had never received treatment before despite pediatric follow up. 12/32 came for a reason other than asthma, such as Food Allergy (3), Urticaria (2), Drug allergy (1), Eczema (1), Allergic Rhinitis (1) and hospitalization due to foreign body aspiration (1). Of the 37/82 children who already had a diagnosis of asthma, 31 had poorly controlled symptoms despite treatment. Reasons for uncontrolled asthma in 21/31 were low doses of Inhaled Corticosteroids or intermittent use, 7/31 had improper inhaler technique and 3/31 had poor adherence to treatment. 9/82 children were referred for severe asthma; 4/9 had improper inhalation technique. Non-adherence to treatment and co-morbid conditions also contributed to persistent symptoms. Patients were treated individually. After 6 months, symptoms were well controlled in 67 children. 3 children were well controlled at the 3 months follow up while 7 children's follow up is pending. 1 child did not return, 1 child followed alternative therapies and 3 were not compliant to our advice.

## Conclusions

Asthma in children is still often underdiagnosed. For correct diagnosis/treatment a detailed clinical history is mandatory and lung function tests should be performed in children with associated comorbidities such as AR. Studies have shown that one demonstration of the inhaler technique is not enough. It is essential to educate clinicians, patients and parents and to promote compliance.

